# Smartphone-Based Intervention Targeting Norms and Risk Perception Among University Students with Unhealthy Alcohol Use: Secondary Mediation Analysis of a Randomized Controlled Trial

**DOI:** 10.2196/55541

**Published:** 2025-02-06

**Authors:** Joseph Studer, John A Cunningham, Elodie Schmutz, Jacques Gaume, Angéline Adam, Jean-Bernard Daeppen, Nicolas Bertholet

**Affiliations:** 1 Addiction Medicine Department of Psychiatry Lausanne University Hospital and University of Lausanne Lausanne Switzerland; 2 Service of Adult Psychiatry North-West Department of Psychiatry Lausanne University Hospital and University of Lausanne Nyon Switzerland; 3 National Addiction Centre Institute of Psychiatry, Psychology and Neuroscience King’s College London London United Kingdom; 4 Center for Addiction and Mental Health Toronto, ON Canada; 5 Department of Psychiatry University of Toronto Toronto, ON Canada

**Keywords:** brief intervention, alcohol use, mechanism of action, mediation analysis, personalized feedback, smartphone app, students, Switzerland, mobile phone, mediation, feedback, student, health risk, drinking, drinker, support, feedback intervention

## Abstract

**Background:**

Many digital interventions for unhealthy alcohol use are based on personalized normative feedback (PNF) and personalized feedback on risks for health (PFR). The hypothesis is that PNF and PFR affect drinkers’ perceptions of drinking norms and risks, resulting in changes in drinking behaviors. This study is a follow-up mediation analysis of the primary and secondary outcomes of a randomized controlled trial testing the effect of a smartphone-based intervention to reduce alcohol use.

**Objective:**

This study aimed to investigate whether perceptions of drinking norms and risks mediated the effects of a smartphone-based intervention to reduce alcohol use.

**Methods:**

A total of 1770 students from 4 higher education institutions in Switzerland (mean age 22.35, SD 3.07 years) who screened positive for unhealthy alcohol use were randomized to receive access to a smartphone app or to the no-intervention control condition. The smartphone app provided PNF and PFR. Outcomes were drinking volume (DV) in standard drinks per week and the number of heavy drinking days (HDDs) assessed at baseline and 6 months. Mediators were perceived drinking norms and perceived risks for health measured at baseline and 3 months. Parallel mediation analyses and moderated mediation analyses were conducted to test whether (1) the intervention effect was indirectly related to lower DV and HDDs at 6 months (adjusting for baseline values) through perceived drinking norms and perceived risks for health at 3 months (adjusting for baseline values) and (2) the indirect effects through perceived drinking norms differed between participants who overestimated or who did not overestimate other people’s drinking at baseline.

**Results:**

The intervention’s total effects were significant (DV: b=–0.85, 95% bootstrap CI –1.49 to –0.25; HDD: b=–0.44, 95% bootstrap CI –0.72 to –0.16), indicating less drinking at 6 months in the intervention group than in the control group. The direct effects (ie, controlling for mediators) were significant though smaller (DV: b=–0.73, 95% bootstrap CI –1.33 to –0.16; HDD: b=–0.39, 95% bootstrap CI –0.66 to –0.12). For DV, the indirect effect was significant through perceived drinking norms (b=–0.12, 95% bootstrap CI –0.25 to –0.03). The indirect effects through perceived risk (for DV and HDD) and perceived drinking norms (for HDD) were not significant. Results of moderated mediation analyses showed that the indirect effects through perceived drinking norms were significant among participants overestimating other people’s drinking (DV: b=–0.17, 95% bootstrap CI –0.32 to –0.05; HDD: b=–0.08, 95% bootstrap CI –0.15 to –0.01) but not significant among those not overestimating.

**Conclusions:**

Perceived drinking norms, but not perceived risks, partially mediated the intervention’s effect on alcohol use, confirming one of its hypothesized mechanisms of action. These findings lend support to using normative feedback interventions to discourage unhealthy alcohol use.

**Trial Registration:**

ISRCTN Registry 10007691; https://doi.org/10.1186/ISRCTN10007691

## Introduction

### Background

Unhealthy alcohol use is the highest risk factor for lowering young adults’ disability-adjusted life years [1]. University students are particularly vulnerable to alcohol-related consequences [2-5] and thus constitute an appropriate target for selective prevention. Brief interventions (BIs) are one approach to reducing unhealthy alcohol use and its related consequences, and they are recommended by the World Health Organization [6], the United States Preventive Services Task Force [7], and the United Kingdom’s National Institute for Health and Care Excellence [8]. The efficacy of BIs, administered using a variety of formats (ie, face-to-face, through the internet, on a computer, through a smartphone app), has been demonstrated among individuals not actively seeking treatment [9,10]. However, the mechanisms underlying the efficacy of BIs are still not completely understood, particularly with web- and smartphone-based BIs. Many of the digital interventions for unhealthy alcohol use that have been found effective were based on personalized normative feedback (PNF) and personalized feedback on risks for health (PFR) [11-13].

### Personalized Normative Feedback and Personalized Feedback on Risks for Health in Brief Interventions

PNF is based on social norms theory, which posits that an individual’s behavior is influenced by their perceptions and beliefs about what is considered normal behavior among other people [2,14-16]. Thus, an individual’s alcohol consumption is impacted by their perceptions of other people’s drinking habits. Previous studies have shown that there is a high prevalence of individuals who overestimate the amount that others drink and that those who overestimate other people’s drinking typically consume more alcohol [17,18]. There are also indications that this relationship may be bidirectional, that is, the more an individual overestimates their peers’ drinking behaviors, the more they drink; and the more an individual drinks, the more they overestimate their peers’ drinking [19]. Therefore, providing accurate information about actual norms, using PNF, is expected to correct an individual’s overestimations of other people’s drinking and encourage them to reduce their alcohol use by adopting drinking behaviors more aligned with actual norms [20]. Digital and in-person BIs that aim to correct an individual’s overestimations of other people’s drinking (using PNF) are considered effective [13]. In BIs that include a PNF component, individuals are typically asked to provide information on their sex, age, personal alcohol consumption, and perceptions of their peers’ alcohol consumption. The PNF then consists of a graphical comparison between the user’s own alcohol use, their peers’ alcohol use, and that of a reference group (eg, individuals of the same age and sex in the general population) [14,20,21]. It has been proposed that using more proximal normative reference groups (eg, gender-specific, age-specific, and group-specific) compared with more distal reference groups (eg, the general population) may enhance the efficacy of PNF [22]. However, this proposition has been challenged. For example, in a randomized controlled trial (RCT) among college students, LaBrie and colleagues [23] compared the efficacy of PNF with different levels of specificity of the reference group (ie, 7 groups with typical same-campus student as a reference with level of specificity based on a combination of gender, race, and Greek affiliation) with a typical same-campus student as a reference. Results indicated that participants receiving PNF with more specific reference groups showed less reduction in alcohol-related outcomes compared with those who received PNF referencing a typical student. In addition, other forms of PNF have been designed to improve motivation, engagement, and adherence. For instance, in 2 studies among college students, the addition of gamified elements (eg, a point-based reward system, norm visualization through visibly connected peers on Facebook [Meta], and elements of chance) was associated with greater reductions in both perceived peer drinking norms and personal alcohol use than standard PNF [24,25].

PFR is based on various theories related to health-protective behavior and emphasizes the significant motivational influence of perceived risk [26-29]. The desire to prevent or minimize negative health outcomes (perceived benefits) is thought to generate greater motivation for self-protection [30]. Consequently, PFR is anticipated to improve an individual’s awareness of risks and motivation for self-protection and to result in reduced alcohol consumption or altered drinking patterns.

Thus, perceived drinking norms and perceived risks for health are expected to serve as mechanisms of action of PNF and PFR interventions and, therefore, they should be tested as mediators for the impact of such interventions on alcohol consumption [30,31]. The large number of RCTs showing the beneficial effects on alcohol outcomes of BIs incorporating PNF or PFR could be interpreted as indirect support for this assumption [23,32-35]. However, since most BIs comprise multiple components, including, but not limited to, PNF and PRF, these studies failed to formally test the mediation of interventions’ effects through perceived norms and risks. Few studies have directly investigated whether perceived norms mediated the effects of face-to-face [36-38] and digital [23,39-43] BIs on alcohol use. These studies were principally conducted among students [23,36-40,43], military personnel, or veterans [41,42]. They showed significant mediation through perceived drinking norms, although the results were not always consistent across outcomes. Furthermore, many of these studies failed to ensure the temporal ordering necessary between intervention, mediator, and outcome required for a mediation analysis (eg, studies by Borsari and Carey [36], Kulesza et al [37], Walters et al [39], and Pedersen et al [42]). Mediation occurs in a causal chain involving 2 causal associations, that is, the intervention influences the mediator and the mediator influences the outcomes, so the intervention must precede the mediator and the mediator must precede the outcome [44]. Concerning risk perception, only 1 study to date has, to the best of our knowledge, investigated whether changes in risk perception mediated the effect of BIs on alcohol use [41], but no significant mediation was observed.

### This Study

We developed a smartphone app designed to reduce alcohol use among students reporting unhealthy alcohol use [45], and we tested its efficacy in an RCT involving university students reporting unhealthy alcohol use, identified by screening. Providing access to the app (vs not providing access) was associated with significantly fewer standard drinks consumed per week and heavy drinking days (HDDs) per month over the 12-month follow-up period [32]. This study aimed to test whether perceived drinking norms and perceived risks for health mediated the effects of providing access to the app. We hypothesized that perceived drinking norms and perceived risks for health at 3 months would partially mediate the BI’s effects on alcohol use at 6 months, controlling for baseline measures. In addition, since PNF is thought to correct individuals’ overestimations of other people’s drinking, it should primarily affect the norms perception of individuals who overestimate other people’s drinking. Thus, our secondary hypothesis was that mediation through norms perception would primarily occur among individuals who overestimate other people’s drinking (ie, moderated mediation). To the best of our knowledge, this is the first study to assess moderated mediation in such a setting.

## Methods

### Overview

This manuscript adheres to the recommendations outlined in A Guideline for Reporting Mediation Analyses (AGReMA) [46]. This study was a secondary analysis of an RCT estimating the effects of providing access to a smartphone app to reduce alcohol use among university students with self-reported unhealthy alcohol use, identified by screening. The results of the primary analysis of this RCT were reported elsewhere [32] in accordance with the CONSORT (Consolidated Standards of Reporting Trials) reporting guidelines [47]. The only deviation from the planned mediation analysis published in the institutional review board–approved protocol is the use of the Mplus software (Muthén & Muthén) instead of SPSS (IBM Corp) with the process macro, although models are equivalent.

### Recruitment and Sample

All participants were recruited in April 2021 at 4 higher education institutions in the Lausanne area in Switzerland (the University of Lausanne, the Swiss Federal Institute of Technology Lausanne, the Hospitality Business School Lausanne, and the University of Applied Sciences and Arts Western Switzerland’s School of Health) and provided their written informed consent to participate. The study’s procedures have been detailed elsewhere [11,32,45]. Each institution promoted the study through its official communication channels (eg, information screens and hallway posters) and student associations’ websites and social media. Students were invited to visit the study’s specially developed website, where they could complete an anonymous questionnaire assessing their eligibility to participate, that is, being a student at the moment of recruitment, being ≥18 years old, owning a smartphone, and screening positive for unhealthy alcohol use (defined as an Alcohol Use Disorders Identification Test–Consumption score ≥4 for males and ≥3 for females [48-50]). The sample size computation was done for the evaluation of intervention effects at 6 months on the primary outcome of the RCT [32], not for the evaluation of the mediation hypothesis. Of the 3714 students who completed the eligibility questionnaire, 2364 were eligible to participate. Baseline assessments were completed by 1770 students, who were subsequently included in the study and were directly randomized in the study website to either the intervention (n=884) or the control group (n=886). This procedure ensured a total concealment of allocation. Follow-up assessments were done electronically, ensuring blinding. Participants were followed up for 12 months until May 2022. This analysis used baseline, 3-month, and 6-month data. Follow-up rates were 96.4% (total: 1706/1770; intervention: 846/884; and control: 860/886) at 3 months and 95.9% (total: 1697/1770; intervention: 846/884; and control: n=851/886) at 6 months. 

### Intervention

The app’s content was based on the existing literature [9,51-55], previous research involving digital interventions conducted by our group [56-60], and input from members of the target population [45]. Its development has been reported elsewhere [11,45]. Briefly, the app comprised 6 modules involving norms perception and risk perception elements.

First, personal feedback on self-reported alcohol consumption, including normative feedback, feedback on the calorific content of the reported consumption, and feedback on health risks. The user’s reported alcohol consumption is compared with the alcohol consumption of people of the same sex and age in Switzerland, with an emphasis on the percentage of people drinking less than the user. This normative feedback is presented for the reported number of drinks per week and the frequency of heavy episodic drinking. Swiss population data were used to generate normative feedback based on the Swiss Health Survey [61] and Addiction Monitoring in Switzerland data [62]. The user also receives an indication of the risks associated with their drinking using specific examples (eg, risk of an accident or violence associated with heavy drinking occasions, risk of addiction, sleep disorders, and cancer associated with chronic heavy drinking). The calorific content of the reported alcohol use is indicated (in kcal), and the total kcal is also presented in “hamburger equivalents” extrapolated to a 3-month period. At the end of the module, the user can choose to set themselves drinking limits by using a link to the goal-setting tool (ie, module 4).

Second, a blood alcohol content computation tool. This module provides a computed estimate of the blood alcohol content reached with the reported consumption and an indication of the risks associated with different levels of blood alcohol content (eg, “with a blood alcohol content of 1 g/L, the risk of a fatal accident is 7 times higher than for 0.5 g/L” and “altered judgement”). The module also computes how long it will take for the alcohol to be eliminated.

Third, a self-monitoring tool. Once the module is activated, the user is invited to report on their drinking daily. The drinking pattern is then presented to the user on a graph, with indications of the recommended drinking limits.

Fourth, a goal-setting tool. This module allows the user to set their drinking limits for 1, 2, 7, or 30 days. Users are then invited to report their drinking daily. They receive a badge when they drink at or below their self-determined drinking limits.

Fifth, a designated driver tool. This module allows user to take pictures of themselves and their friends. The app then randomly picks the picture of the designated sober driver.

Sixth, fact sheets. This module presents fact sheets on alcohol and health (ie, the effects of alcohol on the human body, diseases caused by alcohol, acute and long-term effects of alcohol use on health, addiction, and resources [available treatment options and contacts]). Participants in the intervention group were not given any limitations to their use of the app (they could use it as much or as little as they wished).

### Measures

Follow-up assessments were completed 3 and 6 months after baseline. Email invitations containing a personalized link to the questionnaires were sent out to participants.

#### Outcomes

The primary (ie, weekly DV) and secondary (ie, number of HDDs) outcomes were prespecified in the research protocol [11]. Weekly DV was assessed using a quantity and frequency measure [63]. Drinking frequency was assessed using a question about the average number of days per week on which the individual had used alcohol in the past 30 days. Drinking quantity was evaluated with a question about the average number of standard drinks consumed on drinking days. Pictograms of Swiss standard drinks containing approximately 10-12 grams of pure alcohol were provided as a visual aid. Weekly DVs (ie, the mean number of standard drinks per week over the past 30 days) were the product of drinking frequency and drinking quantity. The number of HDDs was assessed by asking participants about the number of days they had drunk ≥5 drinks for men or ≥4 drinks for women over the past 30 days.

#### Mediators

Perceived norms for weekly DV were assessed by asking participants to report their perceptions of the drinking quantity and frequency of a typical person of their age and sex. Perceived drinking norms were obtained by multiplying normative perceptions of drinking quantity and frequency. For HDDs, perceived norms were measured by asking participants how often a typical person of their age and sex drank 5 or more (for male) or 4 or more (for female) standard drinks on 1 occasion over a 30-day period. Perceived risks for health were assessed by asking participants to report the extent to which they believed that they would be personally at risk of being hurt or falling sick because of their drinking on a 10-point scale (1=no risk and 10=high risk).

#### Covariates

Covariates included age and sex, which were assessed at baseline.

#### Moderator

To determine whether participants overestimated other people’s drinking at baseline, they were asked to estimate the percentage of people of their age and sex who drank more than they did (ie, a subjective estimation from 0% to 100%). Then, the objective percentage of people drinking more than the participant was estimated by comparing the participant’s baseline DV with that of individuals in the Swiss population of the same age and sex, based on the Swiss Health Survey [61] and on Addiction Monitoring in Switzerland data [62]. Finally, the difference between the subjective and objective estimations of other people’s DV was computed. Based on Bertholet et al [17], an overestimation of other people’s drinking (coded 1) was defined as a difference between subjective and objective estimations of their DV of >5 percentage points, whereas no overestimation (coded 0) was defined as a difference of ≤5 percentage points.

### Statistical Analyses

**Figure 1:**
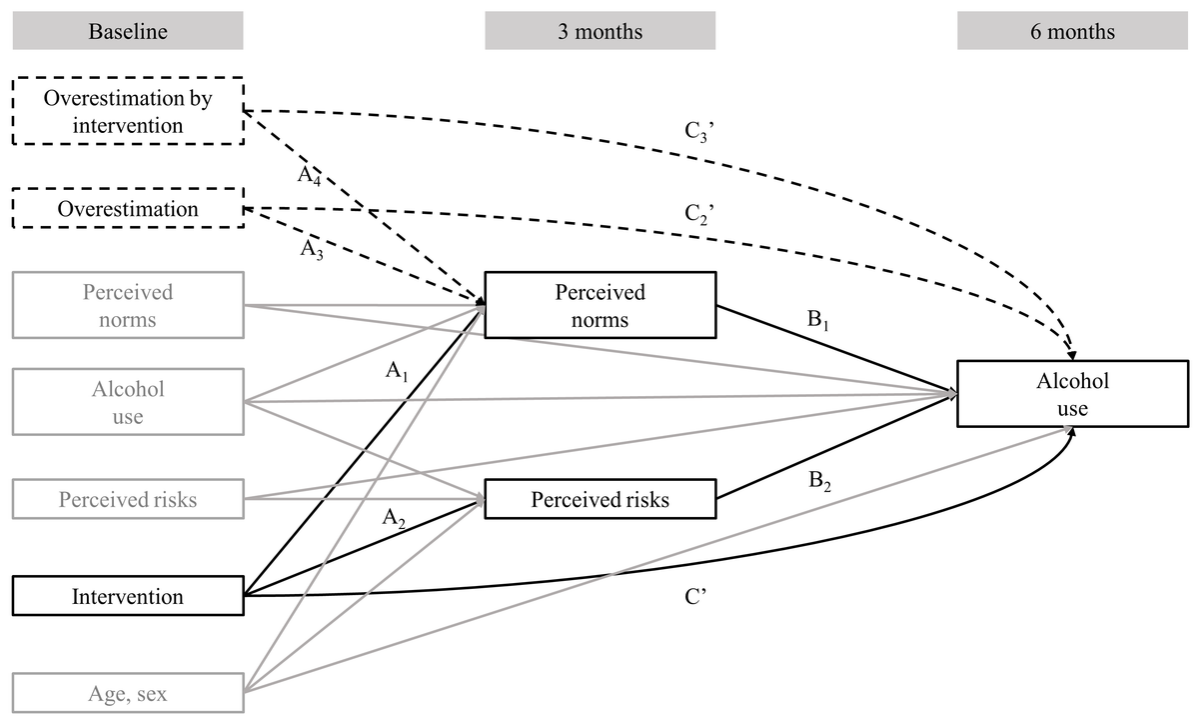
Schematic representation of the structural model of our brief intervention associated with alcohol use, both directly and indirectly through perceived norms and perceived risks at 3 months. Parallel mediation models included all the solid arrows. Moderated mediation models included all the solid and dashed arrows. Only the black arrows were of interest to compute the total, direct, and indirect effects. Gray arrows were only included for adjustment.

Descriptive statistics were calculated to characterize the sample in terms of age, sex, DV, HDDs, perceived DV norms, perceived HDD norms, perceived risks for health, and overestimation of other people’s drinking. We conducted the mediation and moderated mediation analyses to test our hypotheses using Mplus software, version 8.3 [64]. The model tested is depicted in Figure 1. Parallel mediation analysis was used to test the hypotheses that the intervention effect was mediated through perceived drinking norms and perceived risks for health. Separate models were tested for each outcome (ie, DV and HDDs). The outcome at 6 months was regressed on the mediators (ie, perceived drinking norms and perceived risks for health at 3 months), the intervention (vs control), and the baseline values of the outcome and the mediators. The mediators were regressed on the intervention (vs control), their respective baseline values, and the baseline values of the outcomes. All associations were also adjusted for age and sex. This model assumes that intervention causally influences the mediators and that mediators causally influence the outcome. It enabled the estimation of the intervention’s direct effects (ie, adjusted for the mediators) on alcohol use outcomes (path C’) and the 2 specific indirect effects, that is, through perceived norms (A_1_×B_1_) and perceived risks (A_2_×B_2_) at 3 months. The intervention’s total effect (C, ie, not adjusted for the mediators) is the sum of its direct and indirect effects (C=C’+A_1_×B_1_+A_2_×B_2_). If both the indirect and direct associations are significant, then there is evidence for partial mediation. In contrast, if the indirect association is significant and the direct association is 0 or close to 0, then there is evidence of full mediation. Missing values were handled using full information maximum likelihood. Bootstrapping with 10,000 resamples was used to estimate the 95% bootstrap CIs (95% CI) of the parameter estimates. We also computed the ratio of the indirect effects to the total effect as an indication of the indirect effect’s size. As recommended by Hayes [65], this ratio was only computed when total effects were larger than indirect effects and of the same sign.

Moderated mediation was used to test the hypothesis that the intervention effect’s mediation through perceived norms would differ between individuals who overestimated other people’s drinking and individuals who did not overestimate, with mediation primarily expected to be among those individuals overestimating. The moderated mediation model is depicted in Figure 1. Compared with the parallel mediation model described above, the moderated mediation model also tested whether overestimating moderated the A_1_ path (from intervention to perceived norms) and the direct path (C’) from the intervention to the outcome. For this purpose, the overestimation variables and the interaction between intervention and overestimation were included in the model to predict drinking norms at 3 months and drinking outcomes at 6 months. The indirect effects of the intervention through perceived norms were estimated in the moderator’s 2 categories (overestimation and no overestimation). The index of moderated mediation [66], corresponding to the absolute difference between the moderators’ 2 categories’ indirect effects, was also estimated. A sensitivity analysis was conducted using the same (moderated) mediation models on a restricted sample including all participants in the control group (n=886) and participants of the intervention group who accessed module 1 of the app, which included PNF and PFR (n=468). The results of this sensitivity analysis were similar to those of the main analysis. Coefficients were of a similar magnitude and the most significant coefficients in the main analysis remained significant in the sensitivity analysis, with a few exceptions due to lower statistical power. The details of the sensitivity analysis for mediation and moderated mediation models are reported in Tables S1 and S2 in Multimedia Appendix 1.

### Ethical Considerations

The institutional review board protocol was approved by the Human Research Ethics Committee of the Canton of Vaud (number 2018-00560), registered in the ISRCTN registry (ISRCTN10007691), and published before the trial began [11]. The mediation analysis was planned in the Swiss National Science Foundation protocol and the institutional review board–approved protocol, whereas the moderated mediation analysis was not preplanned. All participants received information on the nature of the study, the procedures involved, the expected duration, the potential risks and benefits it may entail, and the financial compensation (gift certificates of up to 50 CHF, equivalent to approximately US $52). They were informed that study participation was voluntary and that they may withdraw from the study at any time without consequences. All participants provided written informed consent before their inclusion in the study. To ensure the confidentiality of data, all data collected on participants were identified with a randomly generated, unique participant identification number. Master lists of participant identification numbers and individually identifiable private information were stored in password-protected computers with restricted access. These lists were available only to senior research staff on this project. The linkage and the direct subject identifiers will be destroyed 10 years after study completion. Subject confidentiality was further ensured by using subject identification code numbers to correspond to treatment data in the computer files. The trial was monitored independently by Lausanne University Hospital’s Clinical Trial Unit according to the International Council on Harmonization of Technical Requirements for Registration of Pharmaceuticals for Human Use Good Clinical Practice.

## Results

### Descriptive Characteristics

**Figure 2:**
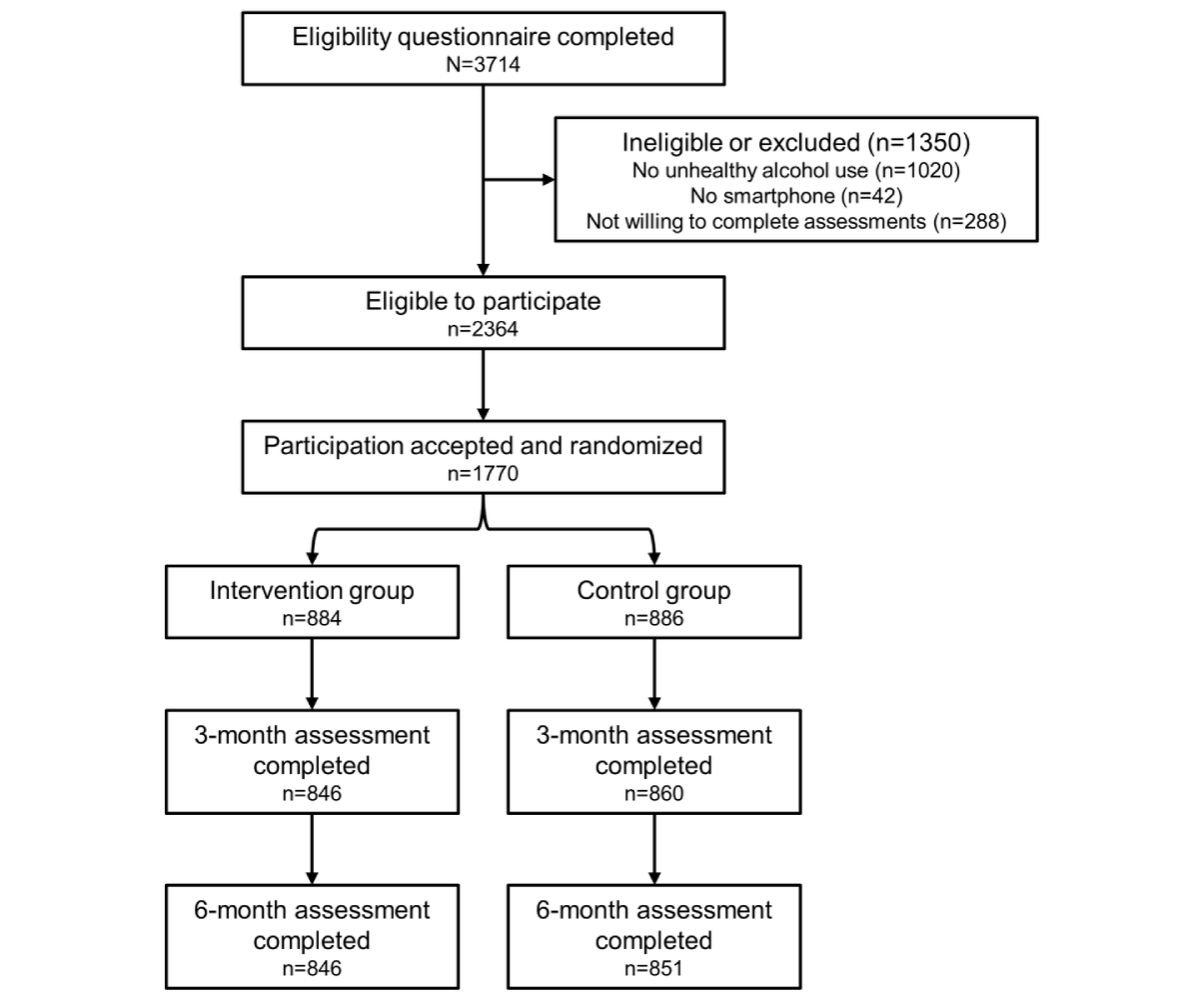
CONSORT flow diagram. CONSORT: Consolidated Standards of Reporting Trials.

Figure 2 contains a CONSORT flow diagram. Participants’ characteristics and alcohol use are reported in Table 1. The mean participant age was 22.35 (SD 3.07) years, and slightly more than half were female. At baseline, they reported a mean weekly DV of 8.59 (SD 8.18) standard drinks and 3.53 (SD 4.02) HDDs in the previous 30 days. Perceived drinking norms were slightly greater than participants’ alcohol consumption, with a mean DV of 8.65 (SD 7.18) standard drinks and 3.67 (SD 2.75) HDDs. On a scale from 1 to 10, participants evaluated the risks to their health associated with their alcohol consumption as relatively low (mean 2.35, SD 1.55). About 78% (1375/1770) of participants overestimated the alcohol consumption of other people of their age and sex in the Swiss population.

**Table 1:** Descriptive characteristics.

Characteristics		Control (n=886)	Intervention (n=884)	Total (N=1770)
**Sex, n (%)**				
	Male	393 (44.4)	419 (47.4)	812 (45.9)
	Female	493 (55.6)	465 (52.6)	958 (54.1)
Age (years), mean (SD)		22.45 (3.27)	22.24 (2.85)	22.35 (3.07)
**Weekly DV^a^, mean (SD)**				
	Baseline	8.25 (7.65)	8.93 (8.66)	8.59 (8.18)
	6 months	7.68 (8.11)	7.11 (6.29)	7.39 (7.26)
**HDDs^b^, mean (SD)**				
	Baseline	3.48 (3.83)	3.58 (4.19)	3.53 (4.02)
	6 months	3.39 (3.65)	3.02 (3.18)	3.21 (3.43)
**Perceived norms of weekly DV, mean (SD)**				
	Baseline	8.72 (6.23)	8.57 (8.03)	8.65 (7.18)
	3 months	9.77 (6.89)	9.02 (6.01)	9.40 (6.48)
**Perceived norms of HDDs, mean (SD)**				
	Baseline	3.73 (2.92)	3.61 (2.57)	3.67 (2.75)
	3 months	4.22 (3.24)	3.96 (3.04)	4.09 (3.14)
**Perceived risks for health, mean (SD)**				
	Baseline	2.36 (1.56)	2.34 (1.54)	2.35 (1.55)
	3 months	2.40 (1.58)	2.43 (1.56)	2.42 (1.57)
**Overestimation, n (%)**				
	No	198 (22.3)	197 (22.3)	395 (22.3)
	Yes	688 (77.7)	687 (77.7)	1375 (77.7)

^a^DV: drinking volume.

^b^HDD: heavy drinking day.

### Mediation Models

The results of our mediation models are reported in Table 2. Regarding DV, the intervention’s total effect (C) was significant (b=–0.85, 95% CI –1.49 to –0.25), indicating a lower DV at 6 months in the intervention group than in the control group. The intervention’s direct effect (C’, ie, its effect adjusted for mediators) was also significant but smaller (b=–0.73, 95% CI –1.33 to –0.16). The intervention’s indirect effect was significant through the drinking norms mediator (b=–0.12, 95% CI –0.25 to –0.03). The intervention was associated with lower estimations of drinking norms at 3 months (b=–0.80, 95% CI –1.36 to –0.22), and estimated drinking norms at 3 months were associated with drinking at 6 months (b=0.15, 95% CI 0.08 to 0.24). The intervention’s indirect effect through estimated drinking norms at 3 months accounted for 14% (ratio of specific indirect effect/total effect=–0.12/–0.85) of the total effect. The intervention’s indirect effect through risk perception was not significant (b=0.00; 95% CI –0.03 to 0.04), and neither was the association between the intervention and perceived risks at 3 months (b=0.02, 95% CI –0.11 to 0.15) or the association between perceived risks at 3 months and DV at 6 months (b=0.20, 95% CI –0.13 to 0.47).

The intervention’s total effect (C) for HDDs was significant (b=–0.44, 95% CI –0.72 to –0.16), indicating fewer HDDs at 6 months in the intervention group than in the control group. The intervention’s direct effect (C’) was also significant but smaller (b=–0.39, 95% CI –0.66 to –0.12). However, the intervention’s indirect effect through drinking norms at 3 months (b=–0.05, 95% CI –0.11 to 0.004) and its indirect effect through risk perception (b=0.00, 95% CI –0.01 to 0.02) were not significant.

**Table 2:** Results of parallel mediation models (all models were adjusted for age, sex, and the baseline values of the outcomes and the mediators).

Path of association		DV^a^ at 6 months		HDD^b^ at 6 months	
		b (SE^c^)	95% CI^d^	b (SE)	95% CI
**Perceived norms as mediator**					
	Intervention to mediator (A)	–0.80^e^ (0.29)	–1.36 to –0.22	–0.26 (0.07)	–0.53 to 0.02
	Mediator to outcome (B)	0.15^e^ (0.04)	0.08 to 0.24	0.19^e^ (0.04)	0.12 to 0.26
	Specific indirect effect	–0.12^e^ (0.06)	–0.25 to –0.03	–0.05 (0.03)	–0.11 to 0.004
	Size of specific indirect effect (ratio of specific indirect effect/total effect)	0.14		0.11	
**Perceived risks as mediator**					
	Intervention to mediator (A)	0.02 (0.07)	–0.11 to 0.15	0.02 (0.06)	–0.11 to 0.15
	Mediator to outcome (B)	0.20 (0.15)	–0.13 to 0.47	0.07 (0.06)	–0.06 to 0.19
	Specific indirect effect	0.00 (0.01)	–0.03 to 0.04	0.00 (0.00)	–0.01 to 0.02
	Size of specific indirect effect (ratio of specific indirect effect/total effect)	0.00		0.00	
Total indirect effect		–0.12^e^ (0.06)	–0.25 to –0.02	–0.05 (0.03)	–0.11 to 0.01
Size of total indirect effect (ratio of total indirect effect/total effect)		0.14		0.11	
Direct effect (C’)		–0.73^e^ (0.30)	–1.33 to –0.16	–0.39^e^ (0.14)	–0.66 to –0.12
Total effect (C)		–0.85^e^ (0.32)	–1.49 to –0.25	–0.44^e^ (0.14)	–0.72 to –0.16

^a^DV: drinking volume.

^b^HDD: heavy drinking day.

^c^SE: robust standard error.

^d^CI: bootstrap confidence interval.

^e^Coefficients are significant based on 95% CI.

### Moderated Mediation Models

**Figure 3:**
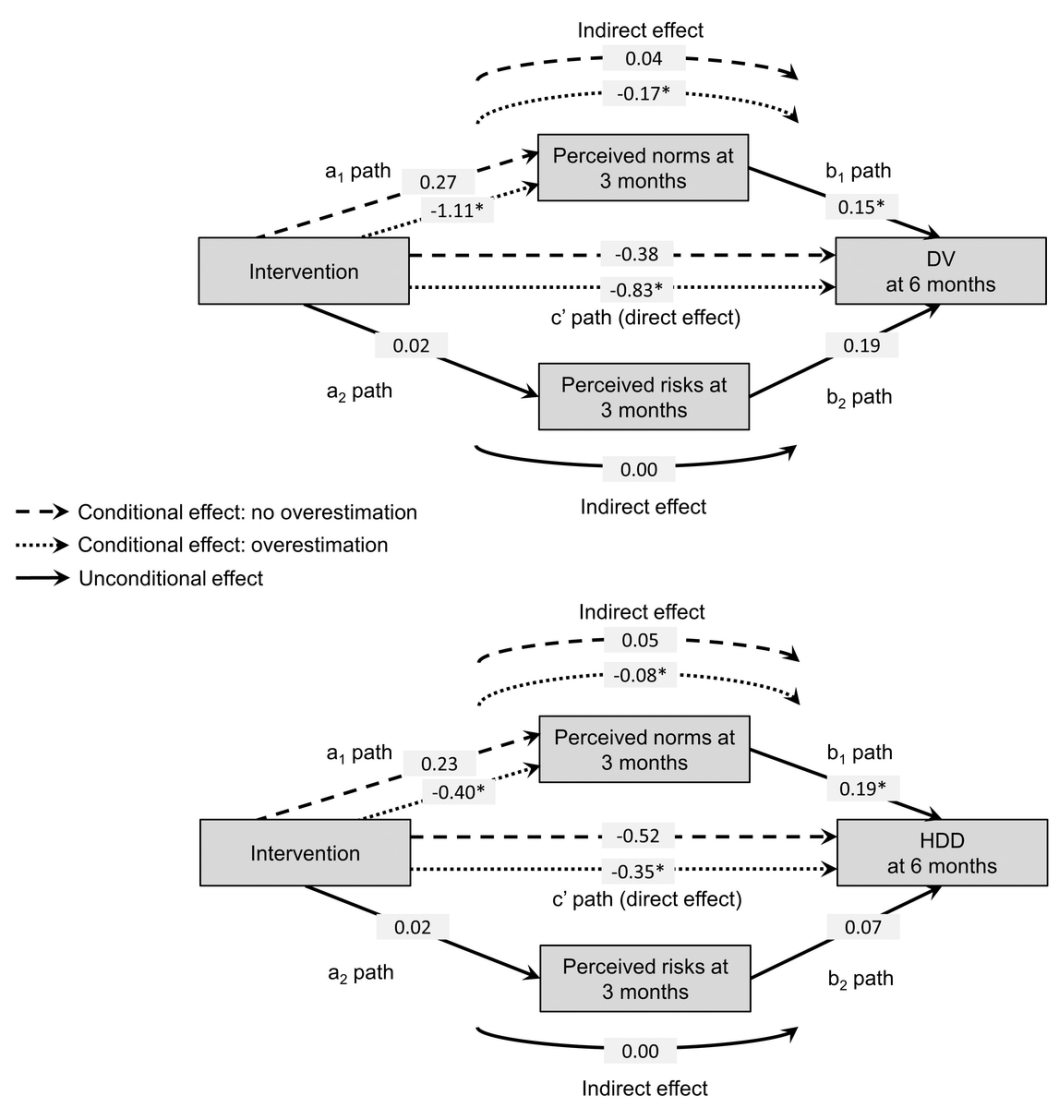
Results of moderated mediation models. An asterisk indicates a coefficient significant based on 95% bootstrap CI. DV: drinking volume; HDD: heavy drinking day.

The results of our moderated mediation models, reported in Table 3 and Figure 3, showed that overestimations of other people’s drinking moderated the association between the intervention and drinking norms at 3 months, in both the DV (b=–1.38, 95% CI –2.82 to –0.03) and HDDs (b=–0.63, 95% CI –1.24 to –0.05) models. The index of moderated mediation was also significant for both DV (b=–0.21, 95% CI –0.49 to –0.01) and HDDs (b=–0.12, 95% CI –0.25 to –0.01), indicating differences in the indirect effects between individuals who overestimated other people’s drinking and those who did not. More specifically, the intervention’s indirect effects on DV and HDDs at 6 months through drinking norms at 3 months was larger (as an absolute value) among individuals who overestimated other people’s drinking (DV: b=–0.17, 95% CI –0.32 to –0.05; HDD: b=–0.08, 95% CI –0.15 to –0.01) than among those who did not (DV: b=0.04, 95% CI –0.14 to 0.26; HDD: b=0.05, 95% CI –0.05 to 0.15), and it was only significant among those who had overestimated. Among individuals who had overestimated other people’s drinking, the indirect effect of the intervention on DV and HDDs through drinking norms accounted for 17% and 19% of the total effect, respectively.

**Table 3:** Results of moderated mediation models (all models were adjusted for age, sex, and the baseline values of the outcomes and the mediators).

Path of association^a^				DV^b^ at 6 months				HDD^c^ at 6 months		
				B (SE^d^)		95% CI^e^		B (SE)		95% CI
Intervention to perceived norms at 3 months (A_1_)				0.27 (0.62)		–0.89 to 1.55		0.23 (0.26)		–0.26 to 0.76
Overestimation to perceived norms at 3 months (A_3_)				0.98 (0.53)		–0.13 to 1.96		0.50^f^ (0.19)		0.12 to 0.88
Overestimation × intervention to perceived norms at 3 months (A_4_)				–1.38^f^ (0.70)		–2.82 to –0.03		–0.63^f^ (0.30)		–1.24 to –0.05
Perceived norms at 3 months to outcome at 6 months (B_1_)				0.15^f^ (0.04)		0.08 to 0.24		0.19^f^ (0.04)		0.12 to 0.26
Intervention to outcome at 6 months (C’)				–0.38 (0.59)		–1.55 to 0.77		–0.52 (0.29)		–1.09 to 0.04
Overestimation to outcome at 6 months (C_2_’)				0.02 (0.48)		–0.97 to 0.93		–0.22 (0.26)		–0.77 to 0.28
Overestimation × intervention to outcome at 6 months (C_3_’)				–0.46 (0.67)		–1.76 to 0.83		0.18 (0.33)		–0.47 to 0.82
**Conditional effects of the intervention**										
	***No overestimation***									
		Indirect effect (through perceived norms at 3 months)	0.04 (0.10)		–0.14 to 0.26		0.05 (0.05)		–0.05 to 0.15	
		Size of the indirect effect (ratio of indirect effect/total effect)	—^g^				—			
		Direct effect	–0.38 (0.59)		–1.56 to 0.77		–0.52 (0.29)		–1.09 to 0.04	
		Total effect	–0.34 (0.60)		–1.52 to 0.87		–0.48 (0.29)		–1.04 to 0.08	
	***Overestimation***									
		Indirect effect (through perceived norms at 3 months)	–0.17^f^ (0.07)		–0.32 to –0.05		–0.08^f^ (0.03)		–0.15 to –0.01	
		Size of the indirect effect (ratio of indirect effect/total effect)	0.17				0.19			
		Direct effect	–0.83^f^ (0.34)		–1.50 to –0.18		–0.35^f^ (0.16)		–0.66 to –0.04	
		Total effect	–1.00^f^ (0.36)		–1.72 to –0.31		–0.42^f^ (0.17)		–0.74 to –0.10	
	Index of moderation		–0.21^f^ (0.12)		–0.49 to –0.01		–0.12^f^ (0.06)		–0.25 to –0.01	

^a^Indirect effects mediated by risk perception were estimated in the model but not reported.

^b^DV: drinking volume.

^c^HDD: heavy drinking day.

^d^SE: robust standard error.

^e^CI: bootstrap confidence interval.

^f^Coefficients are significant based on 95% CI.

^g^Not applicable.

## Discussion

### Principal Findings

This study’s results showed that the effects of a smartphone-based intervention to reduce alcohol use were partially mediated by perceived drinking norms but not by its perceived risks. Specifically, the intervention was significantly associated with lower perceived drinking norms at 3 months, and perceived drinking norms at 3 months were associated with weekly DV at 6 months. The same pattern of association was observed for HDDs, although the indirect effect mediated by perceived drinking norms was not significant. This finding is in line with results from previous studies showing that perceived norms mediated the effects of face-to-face [36-38] and digital [39-43] BIs on alcohol use.

Furthermore, the intervention’s indirect effects on weekly DV and HDDs at 6 months through perceived drinking norms at 3 months, were only significant among individuals who had overestimated other people’s alcohol use. This finding is consistent with the theoretical bases of PNF, as it is primarily expected to influence an individual’s drinking behaviors by correcting their overestimation of other people’s drinking and encouraging them to adopt drinking behaviors that are more aligned with actual norms [20]. Thus, for individuals who did not overestimate other people’s drinking, PNF is not expected to affect their perceptions of norms, which was consistently observed throughout this study.

Taken together, these results confirmed one of the hypothesized mechanisms of action of BIs [20]. They extended previous studies supporting the use of PNF in both internet-based [39-42] and face-to-face [36,37] BIs and lent support to its application in smartphone-based BIs to lower unhealthy alcohol use. PNF appeared to partially mediate the intervention’s effects when individuals overestimated other people’s drinking. As such, providing participants with information that their drinking was heavier than the norm had a conditional impact on those who misperceived their drinking with respect to other people’s drinking. Nevertheless, we found no evidence of full mediation. Among individuals who overestimated other people’s drinking, the mediation of perceived drinking norms accounted for 17% and 19% of the intervention’s effect on DV and HDDs, respectively. Although this accounts for only a limited portion of the intervention’s total effect, these results support the use of normative feedback interventions as an intervention option for secondary prevention interventions aiming at decreasing alcohol-related harm in the population. This type of digital intervention has the potential to be scaled up and reach a wide audience at a low cost, contributing to public health efforts in mitigating alcohol-related harm. It is among the NICE (National Institute for Health and Care Excellence) recommended options to address unhealthy alcohol use [67]. The remaining portion of the intervention’s effect not explained by drinking norms leaves room for other mechanisms of action contained within the intervention. The app, developed previously, included other modules that may have played a role in the intervention’s effects, such as setting drinking goals and monitoring one’s own alcohol use [45], but their hypothesized mechanisms of action were not assessed or tested. Also, using the app may have led participants to reassess their alcohol use and their relationship with alcohol through other mechanisms not evaluated here. Assessment reactivity may also have exerted an effect on the participants in both study groups, but because the intervention group’s participants had the ability to repeatedly assess their alcohol use, they may have been more disposed to changing their drinking behavior for this reason [68]. This warrants further studies to better understand the mechanisms of action of screening and BIs. Nonetheless, this type of intervention should be seen as one contributing piece in a comprehensive public health approach including, as recommended by the SAFER (Strengthen restrictions on alcohol availability; Advance and enforce drink driving countermeasures; Facilitate access to screening, brief interventions, and treatment; Enforce bans or comprehensive restrictions on alcohol advertising, sponsorship, and promotion; Raise prices on alcohol through excise taxes and pricing policies) initiative [69], contextual and structural prevention measures, such as restrictions on the availability and ban on advertising.

The lack of any mediation effect by perceived risks has to be interpreted in light of the study population. In line with results showing that risk perceptions for health related to alcohol use are lower in younger than in older adults [70], perceived risks (and likely actual risks) were low in our sample of young adults. Using the same question, the mean risk perception in our sample was approximately half of that reported in a North American sample of older participants reporting unhealthy drinking (mean age 37.9, SD 9.7 years) [71]. This suggests that young adults may be less concerned by the potential alcohol-related consequences on health than middle-aged and older adults because the potential consequences seem so far away. Thus, the role of perceived risks in BIs may depend on the age of the individuals involved. One alternative explanation for the lack of mediation by perceived risks is that digital interventions may not provide the same level of interaction, emotional engagement, and immediate clarification as face-to-face interventions, which can make PFR more impactful. In digital formats, the static nature of risk messages may reduce their perceived relevance and impact, making it harder to convey the seriousness of health risks compared with a more dynamic and responsive face-to-face setting. Further studies should replicate these analyses in other populations and explore interventions that deliver more dynamic and personalized risk feedback, such as using teleconsultations or adaptive messaging, to better engage users and enhance the perceived relevance of health risks.

### Strengths and Limitations

This study had some limitations related to the use of self-reported measures, which can be susceptible to underreporting and social desirability bias, notably when norms are provided. Nonetheless, we used standardized measures, and the intervention’s effects in the main trial were consistent for both the outcomes that provided us with normative feedback and the outcomes that did not [32]. The study was conducted among students, who may have been more able to deal with the complex cognitive process of comparing one’s own drinking with other people’s drinking, and better able to infer a percentage of people drinking more than oneself. Interventions with personalized feedback may be less effective in nonacademic settings [72]. Thus, these results need to be replicated in other settings. In addition, we used national drinking norms based on sex. Because of biological differences between the sexes in alcohol pharmacokinetics and alcohol-related risks and consequences [73], providing information based on sex is relevant. Nonetheless, some participants may consider gender norms more relevant to them and may not find sex norms adequate. As the perceived validity of norms is crucial to normative feedback being considered relevant, and as data on drinking norms by gender are currently lacking, this will likely be a challenge for future interventions. Furthermore, our sample of students with unhealthy drinking habits may be surrounded by individuals who consume more alcohol than those in the general population. As a result, our participants might have been skeptical of the information provided by PNF, which is based on nationally representative data for individuals of the same age and sex, as it may not reflect their personal experiences. Promising alternative approaches, such as using dynamic norms (as opposed to static norms) [74,75] or emphasizing deviant versus normative behaviors depending on the context and prevalence of the behavior [76], have been proposed. Evaluating their effectiveness in future interventions is warranted. The distribution of the HDD and DV outcomes, as well as perceived drinking norms, were right-skewed, which may violate the normality assumption of linear regression. To mitigate this issue, bootstrap resampling was used to provide accurate CIs without relying on parametric assumptions [77].

The study has some notable strengths. The mediation hypotheses were preplanned, and outcomes and mediators were assessed sequentially, ensuring proper temporal ordering so that the intervention occurred before the mediators, which acted before the outcomes. The mediators were also measured at baseline, allowing for the adjustment of baseline values. We were able to conduct a mediation analysis in an RCT that demonstrated the intervention’s beneficial impact. Because of the nature of the mediators studied, they could be assessed in both groups with a limited risk of assessment reactivity.

### Conclusions

This study’s findings lend support to 1 of the 2 hypothesized mechanisms of action of the digital BI tested—the mechanism of PNF but not the mechanism of feedback on risks. This supports the use of PNF in similar interventions. Nonetheless, only a limited portion of the intervention’s effects could be explained. Further research is therefore needed to identify other mechanisms of action of digital BIs.

## Appendix

Multimedia Appendix 1 Supplementary tables.

## Abbreviations

AGReMA: A Guideline for Reporting Mediation Analyses
BI: brief intervention
CONSORT: Consolidated Standards of Reporting Trials
DV: drinking volume
HDD: heavy drinking day
NICE: National Institute for Health and Care Excellence
PFR: personalized feedback on risks for health
PNF: personalized normative feedback
RCT: randomized controlled trial
SAFER: Strengthen restrictions on alcohol availability; Advance and enforce drink driving countermeasures; Facilitate access to screening, brief interventions, and treatment; Enforce bans or comprehensive restrictions on alcohol advertising, sponsorship, and promotion; Raise prices on alcohol through excise taxes and pricing policies

## Data Availability

The data analyzed in this study are available upon reasonable request to the corresponding author. Access will be granted following an approved data-sharing agreement and in compliance with ethical and institutional guidelines.
