# Supplementary material for: Smartphone-Based Intervention Targeting Norms and Risk Perception Among University Students with Unhealthy Alcohol Use: Secondary Mediation Analysis of a Randomized Controlled Trial
Source: J Med Internet Res. 2025 Feb 6;27:e55541. doi: 10.2196/55541 (PMC11843063; doi:10.2196/55541)
Supplement: Multimedia Appendix 1 [file jmir_v27i1e55541_app1.docx]

Multimedia Appendix

Table S1. Results of parallel mediation models (sensitivity analysis)

|  | **DV at 6 months** | | | **HDD at 6 months** | | |
| --- | --- | --- | --- | --- | --- | --- |
|  | **b** | **SE** | **95% CI** | **b** | **SE** | **95% CI** |
| **Perceived norms as mediator** |  |  |  |  |  |  |
| **Intervention -> mediator (A)** | *-0.85* | 0.34 | -1.50; -0.19 | *-0.35* | 0.16 | -0.67; -0.04 |
| **Mediator -> outcome (B)** | *0.14* | 0.05 | 0.06; 0.25 | *0.19* | 0.04 | 0.11; 0.28 |
| **Specific indirect effect** | -*0.12* | 0.06 | -0.26; -0.02 | *-0.07* | 0.04 | -0.14; -0.01 |
| **Size of specific indirect effect** | 0.14 | | | 0.13 | | |
|  |  | | |  | | |
| **Perceived risks as mediator** |  |  |  |  |  |  |
| **Intervention -> mediator (A)** | 0.02 | 0.08 | -0.11; 0.15 | 0.03 | 0.08 | -0.13, 0.18 |
| **Mediator -> outcome (B)** | 0.21 | 0.18 | -0.13; 0.47 | 0.07 | 0.08 | -0.07; 0.22 |
| **Specific indirect effect** | 0.00 | 0.02 | -0.03; 0.04 | 0.00 | 0.01 | -0.02; 0.02 |
| **Size of specific indirect effect** | 0.00 | | | 0.00 | | |
|  |  |  |  |  |  |  |
| **Total indirect effect** | *-0.12* | 0.06 | -0.26; -0.003 | *-0.07* | 0.04 | -0.15; -0.003 |
| **Size of total indirect effect** | 0.14 | | | 0.13 | | |
| **Direct effect (C’)** | *-0.76* | 0.32 | -1.40; -0.14 | *-0.47* | 0.15 | -0.77; -0.17 |
| **Total effect (C)** | *-0.88* | 0.33 | -1.55; -0.23 | *-0.53* | 0.16 | -0.84; -0.23 |

Note. CI = bootstrap confidence interval; SE = robust standard error; DV = drinking volume; HDD = heavy drinking days. Coefficients in italics are significant based on 95%CI. All models were adjusted for age, sex, and the baseline values of the outcomes and the mediators.

Table S2. Results of moderated mediation models (sensitivity analysis)

|  | **DV at 6 months** | | | **HDD at 6 months** | | |
| --- | --- | --- | --- | --- | --- | --- |
|  | **B** | **SE** | **95%CI** | **B** | **SE** | **95%CI** |
| **Intervention -> perceived norms at 3 months (A_1_)** | 0.52 | 0.83 | -1.05; 2.27 | 0.13 | 0.31 | -0.46; 0.76 |
| **Overestimation -> perceived norms at 3 months (A_3_)** | 0.57 | 0.46 | -0.37; 1.46 | *0.52* | 0.19 | 0.15; 0.90 |
| **Overestimation * Intervention -> perceived norms at 3 months (A_4_)** | *-1.77* | 0.91 | -3.66; -0.05 | -0.62 | 0.36 | -1.36; 0.07 |
| **Perceived norms at 3 months -> outcome at 6 months (B_1_)** | *0.14* | 0.05 | 0.06; 0.24 | *0.19* | 0.04 | 0.11; 0.28 |
| **Intervention -> outcome at 6 months (C')** | -0.47 | 0.66 | -1.77; 0.83 | -0.42 | 0.34 | -1.09; 0.24 |
| **Overestimation -> outcome at 6 months (C_2_')** | -0.02 | 0.49 | -1.02; 0.93 | -0.19 | 0.27 | -0.72; 0.32 |
| **Overestimation * Intervention -> outcome at 6 months (C_3_')** | -0.37 | 0.75 | -1.83; 1.10 | -0.06 | 0.38 | -0.82; 0.69 |
|  |  |  |  |  |  |  |
| **Conditional effects of the intervention** |  |  |  |  |  |  |
| **No overestimation** |  |  |  |  |  |  |
| **Indirect effect (through perceived norms at 3 months)** | 0.07 | 0.12 | -0.15; 0.38 | 0.02 | 0.06 | -0.09; 0.15 |
| **Size of the indirect effect** | - |  |  | - |  |  |
| **Direct effect** | -0.47 | 0.66 | -1.77; 0.83 | -0.42 | 0.34 | -1.09; 0.24 |
| **Total effect** | -0.40 | 0.68 | -1.74; 0.94 | -0.40 | 0.34 | -1.06; 0.26 |
| **Overestimation** |  |  |  |  |  |  |
| **Indirect effect (through perceived norms at 3 months)** | *-0.18* | 0.07 | -0.36; -0.06 | *-0.09* | 0.04 | -0.19; -0.02 |
| **Size of the indirect effect** | 0.18 |  |  | 0.16 |  |  |
| **Direct effect** | *-0.85* | 0.36 | -1.58; -0.16 | *-0.48* | 0.17 | -0.83; -0.15 |
| **Total effect** | *-1.02* | 0.39 | -1.81; -0.29 | *-0.57* | 0.18 | -0.93; -0.23 |
| **Index of moderation** | *-0.25* | 0.16 | -0.65; -0.01 | -0.12 | 0.07 | -0.28; 0.01 |

Note. CI = bootstrap confidence interval; SE = robust standard error; DV = drinking volume; HDD = heavy drinking days. Indirect effects mediated by risk perception were estimated in the model but not reported. Coefficients in italics are significant based on 95%CI. All models were adjusted for age, sex, and the baseline values of the outcomes and the mediators.
